# Executive Functioning and Learning Skills of Adolescent Children Born at Fewer than 26 Weeks of Gestation

**DOI:** 10.1371/journal.pone.0151819

**Published:** 2016-03-21

**Authors:** A. Farooqi, M. Adamsson, F. Serenius, B. Hägglöf

**Affiliations:** 1 Institute of Clinical Sciences, Pediatrics, University of Umeå, Umeå, Sweden; 2 Department of Women's and Children's Health, Uppsala University, Uppsala, Sweden; 3 Institute of Clinical Sciences, Child and Adolescent Psychiatry, University of Umeå, Umeå, Sweden; Birkbeck College, UNITED KINGDOM

## Abstract

**Aims:**

To assess the cognitive and behavioral aspects of executive functioning (EF) and learning skills in extremely preterm (EPT) children compared with term control children aged 10 to 15 years.

**Methods:**

A total of 132 of 134 (98% of all eligible survivors) EPT children born at the 2 Swedish regional tertiary care centers from 1992 to 1998 (mean age = 12 years, mean birth weight = 718 g, and mean gestational age = 24.4 weeks) and 103 matched term controls were assessed. General intelligence was assessed using the Wechsler Intelligence Scale for Children (WISC-III-R), and cognitive aspects of EF were analyzed using EF-sensitive subscales of the WISC-III-R and Tower test of the Delis-Kaplan Executive Function Scale (D-KEFS). Behaviors related to EF and learning skills were assessed using the Five to Fifteen questionnaire, which is a validated parent and teacher instrument. Academic performance in school was assessed by teachers’ responses on Achenbach’s Teachers Report Form. Analyses performed included multivariate analyses of covariance (ANCOVA and MANCOVA) and logistic regression analyses.

**Results:**

The EPT children displayed significant deficits in cognitive aspects of EF compared with the controls, exhibiting decreases on the order of 0.9 SD to 1.2 SD for tasks of verbal conceptual reasoning, verbal and non-verbal working memory, processing speed and planning ability (P <0.001 for all). After excluding the children with major neurosensory impairment (NSI) or a Full Scale intelligence quotient (FSIQ) of < 70, significant differences were observed on all tests. Compared with controls, parents and teachers of EPT children reported significantly more EF-related behavioral problems. MANCOVA of teacher-reported learning skills in children with FSIQ >70 and without major NSI revealed no interactions, but significant main effects were observed for the behavioral composite executive function score, group status (EPT vs control) and FSIQ, for which all effect sizes were medium to large. The corresponding findings of MANCOVA of the parent-reported learning skills were very similar. According to the teachers’ ratings, the EPT children were less well adjusted to the school environment.

**Conclusion:**

EPT children born in the 1990s who received active perinatal care are at an increased risk of executive dysfunction, even after excluding children with significant neurodevelopmental disabilities. Even mild to moderate executive dysfunctions has a significant impact on learning skills. These findings suggest the need for timely interventions that address specific cognitive vulnerabilities and executive dysfunctions.

## Introduction

Active perinatal care and advances made in the intensive care of extremely preterm infants (EPT, ≤ 25 gestational weeks) have markedly improved survival and lowered the gestational age of viability [1–3]. While over the past two decades the prevalence of severe neurodevelopmental disabilities has remained relatively stable [4,5], there is growing concern that many school-aged children who are born extremely premature with overall intelligence within the normal range have an increased risk of mild to moderate neurocognitive deficits, such as motor impairments, academic underachievement, and behavioral problems [6]. These “low severity/high prevalence” dysfunctions [7] may occur in more than 40% of EPT children [8], but long-term outcome studies in the early adolescence of children born EPT are rare [9–11], especially for infants born after active perinatal care.

There is evidence of impairment in executive functioning (EF) in EPT children of elementary or middle school age [12–18]. EF has been considered to be of the crucial processes related to academic and behavioral problems in preterm or EPT children [19–24]. A commonly used definition of EF refers to interrelated neurocognitive processes that are important for purposeful, goal-directed behavior [25]. There is an ongoing debate on whether EF is a unitary construct or whether it is a set of different functions. In a developmental perspective, three major components of EF have been described, namely inhibition, working memory and shifting [26]. EF gradually develops during childhood and adolescence. The ability to inhibit and control cognitive, behavioral and emotional stimuli and processes is considered by some as fundamental for EF. Working memory is the ability to maintain and interpret information over the short term, and shifting involves the ability to shift between mental states, rules, or tasks, generating different solutions for a problem, and developing strategies to reach a goal [27].

The aim of this study was threefold: 1) to examine the nature, frequency and severity of impairments in executive functions and learning skills in EPT children (mean age of 12 years) who were born at 25 weeks of gestation or fewer in the 1990s and received active perinatal care; 2) to assess whether differences in EF and learning skills are present among EPT children without major neurosensory impairment (NSI) and with a full-scale intelligence quotient (FSIQ) of above -2 SD; and 3) to evaluate the relationships of EF with learning skills and academic attainment. We hypothesized that EPT children have impairments in EF and that mild to moderate deficits have a significant impact on learning skills.

## Methods

### Participants and procedure for recruitment

The study population included survivors among infants born at 23–25 weeks of gestation at the university hospitals of Uppsala and Umea in Sweden between January 1992 and December 1998. These two University hospitals are perinatal referral centers in the northern region of Sweden, with neonatal intensive care units serving the Uppsala region and the northern region of Sweden. During the study period, a total of 261 infants were born at 23–25 weeks of gestation. Of these 261 infants, 213 were born alive at the University hospitals of Umeå and Uppsala. Of these 213, 140 survived to be discharged home. All EPT (23–25 weeks) infants born during the study period were given life support at birth. There were no delivery room deaths, and the survival to discharge home was 66% in this cohort [3]. The short-term outcomes of this cohort have been published previously in a few reports [3,28]. The identification of EPT subjects and other methodological details have been presented elsewhere [29] and will be briefly repeated here. Of the 140 infants who survived after being discharged home, 6 (4.5%) died in the post-neonatal period, with all deaths occurring in the first year of life. In total, 134 infants were identified at 10 to 15 years of age. All of these individuals were eligible for our study. Two children/families refused to participate. At a mean age of 12 years (range 10.1–15.9 years), 132 of 134 survivors (98%) were available for assessment.

Written informed consent was obtained from the caretakers. In cases involving two caretakers, which were commonly encountered, both caretakers had to give their written consent for their child’s participation in the study. An information letter was sent to the children along with a letter to the caretakers asking for their permission to participate in the study. Once the EPT and control families consented to participate in the study, they were contacted by the research coordinator, who explained the assessment and filled out questionnaires. With the parents’ permission, questionnaires were sent to the child's class teacher along with a letter with the relevant instructions for filling out the questionnaires. Questionnaires from all respondents were returned to the study coordinator at the University of Umeå. Two reminders were sent to non-respondents, and when possible, an approach was also made by telephone. Missing data from the returned questionnaires were followed up on in the same way.

The control group was recruited from the national Swedish birth register by selecting a term child of normal birth weight born at the same hospital as and at the closest time (± 7 days) to an EPT child. Six matched control participants were identified for each EPT child, and the parents of the first child on the list were approached. If participation was declined, then the parents of the second child were then approached, and if necessary, additional parents were approached until a control participant agreed to participate or until the pool of 6 children was depleted. The recruitment of controls was a difficult process, which is why only 103 (78%) control children were recruited as opposed to one control for each EPT child as initially planned. The control group was approached and examined in a manner identical to that of the index group.

This study was approved by the Ethical Review Board of Umeå (ERB), SE 90187, Sweden. Umeå ERB is one of six institutional review boards in Sweden. According to the ethical practice and rules of the ERB system in Sweden, the following documents were approved before initiation of the study: the information letters to the parents, children and teachers, the consent forms and research plan, all questionnaires used in the study, the CVs of the researchers and approval certificates from the heads of the clinical departments and from heads of the University clinics (Institute of Clinical Sciences, Department of Pediatrics and Department of Child and Adolescent Psychiatry, Umeå, Sweden). All consent forms are kept together at the University Hospital of Umeå (Umeå University) under the code key of the study.

### Procedures

The subjects were seen for a half-day session by trained psychologists. These assessors were unaware of the neonatal courses of the children.

### Outcome measures

EF is a multidimensional construct involving elements that are cognitive and behavioral in nature. Cognitive elements of EF were assessed by a battery of tests sensitive to specific executive functions [30,31]. In addition, parents and the teachers of participants filled out the questionnaires regarding the behaviors related to EF and learning skills [32,33], and teachers filled out an additional questionnaire (Achenbach’s Teacher Report Form (TRF)) to assess the child’s adaptive functioning, including academic performance [34].

### General intelligence

The Wechsler Intelligence Scale for Children (WISC-III-R), Swedish edition (Wechsler 1999) [31], was used to assess general intelligence. The 13 core subsets were administered and combined to determine the FSIQ.

### Cognitive assessment of EF

We selected 6 core subtests from the WISC-III-R [31] that have been shown to be related to EF, namely Similarities, Digit span, Block design, Picture arrangement, Coding, and Arithmetic. These subtests adequately assess the 3 fundamental cognitive aspects of EF: inhibition, working memory and shifting strategy [26]. We briefly describe these 3 cognitive aspects of EF according to the model proposed by Best and Miller [26] as follows.

*Inhibition* refers to the ability to resist impulses to engage in extraneous behavior. Children with difficulties in inhibition have impaired impulse control and often initiate tasks without listening to instructions (e.g., Attention Deficit /Hyperactivity (ADHD) children)). They typically score poorly on the *similarities subtest (31)*

*Working memory* relates to the ability to actively retain information in the mind during the time that it takes to simultaneously implement a complex cognitive task. *The Coding*, *Arithmetic and Digit span subtests* [31] capture these executive abilities

*Shifting* refers to the ability to initiate a goal-directed behavior, i.e., the production of ideas and the shaping of strategies. The child initiates an activity or several activities to achieve a target goal, and flexibility is required to effectively change and adapt to reach the goal. This flexibility requires good planning skills. The *Block design and Picture arrangement* subtests [31] assess the cognitive ability of shifting.

### WISC-III-R subtests assessing cognitive aspects of EF

#### Similarities

The Similarities subtest assesses verbal conceptual reasoning, which refers to the ability to identify the concept(s) linking words or themes. In this test, the participant is presented with a series of word pairs (e.g., wheel/ball, telephone/radio, or ice/steam), and she or he must identify and explain the common link between each word pair. Each response is scored 2-1-0 according to the quality of the response.

#### Digit span

The Digit span subtest assesses verbal memory, which is the ability to store and process verbal information (auditory memory). It has 2 parts: forward digit span, in which the participant is required to repeat verbatim a given series of numbers, and backward digit span, in which the child is asked to repeat a sequence of numbers in the reverse direction. The number sequences increase from 2 to 9 digits for each sequence. The test is stopped when the child fails both trials of a sequence length.

#### Coding

The Coding subtest is a measure of processing speed that assesses concentration, working memory and visual motor coordination. It requires good eye-hand coordination. In this test, the child transcribes a digit-symbol code. The task is time-limited, with bonuses given for speed.

#### Arithmetic

The Arithmetic subtest assesses the ability to focus attention on a task, short-term memory, the ability to perform simple calculations, and freedom from distraction.

#### Block design

The Block design subtest assesses spatial conceptualization, perceptual organizational ability, and nonverbal reasoning skills. The child is asked to create specific 2-dimensional geometric designs using colored blocks (cubes). This test consists of 12 designs, each with an increasing number of geometric cubes with increasing difficulty, and it is discontinued after 2 failures. Each design has a time limit and bonus points.

#### Picture arrangement

The Picture arrangement subtest assesses visual reasoning and logical thinking and involves the identification of a sequence of steps necessary to attain a specific goal. It consists of 14 scenarios in which the child is presented with a set of colorful pictures in a mixed-up order that requires rearrangement into a logical story.

### Tower test of Delis-Kaplan Executive Function Scale (D-KEFS)

The D-KEFS Tower test [30] requires multiple fundamental cognitive skills and higher-level executive functions. Key fundamental abilities measured by this test include visual attention and visual spatial skills. Executive functions assessed by this test include the following:

*Spatial planning*, or the ability to mentally visualize the outcome of making 2 or moves before they are made;

*Rule learning*, or the ability to acquire heuristic strategies for building towers using the fewest numbers of moves possible (e.g., the ability to learn the best method for clearing a small disk off of a peg to place a bigger disk on the peg);

*Inhibition*, or the ability to refrain from impulsive behavior and to use trial and error to formulate spatial plans or to learn effective problem-solving strategies; and

*Establishing and maintaining cognitive set*, or the capacity to learn the instructional rules to perform a task and to apply those rules consistently while solving each problem.

The overall performance on all the above-mentioned tests for the cognitive components of EF (Similarities, Block design, Picture arrangement, Arithmetic, Coding, and D-KEFS Tower test) was judged according to the age-standard scores, with a mean of 10 (SD: 3).

### Behaviors related to EF

To assess the behavioral parameters related to EF, we used the following parts of the parent and teacher Five to Fifteen (FTF) questionnaire [32,33] that assess the attention, hyperactivity/impulsivity, hypoactivity, and planning/organizing subdomains as well as the working memory domain: 1) Attention (9 items), which assesses the ability to pay attention and to concentrate on various tasks and activities; 2) Hyperactivity/impulsivity (9 items), which assesses the tendency to become too active and includes terms such as ‘fidgets with hands or feet’ and ‘often interrupts or intrudes on others’; 3) Hypoactivity (4 items), which refers to the tendency to become too passive and includes terms such as ‘difficulty getting started’ and ‘seems slow, inert or lacking energy’; 3) Planning/organizing (3 items), which assesses items related to the ability to plan and organize and includes terms such as ‘difficulty understanding consequences of own actions’, ‘difficulty planning and preparing for tasks’ and ‘difficulty completing sequential tasks’; and 4) Working memory (11 items), which contains statements mainly pertaining to short-term memory. All items of the EF subdomains for the attention, impulsivity/hyperactivity, hypoactivity, planning/organizing and working memory domains of the parent and teacher FTF were collapsed into a primary Executive Function Composite Score (EFCS) domain.

#### Learning skills

The learning skills domain of the teacher and parent FTF [32] assesses responses to 29 items related to the child’s learning ability in school subjects, such as math, reading and writing, as well as responses to items related to daily learning ability and coping in learning. The learning skills domain is further organized into 4 subdomains: reading/writing (8 items), math (5 items), general learning (6 items) and coping in learning (10 items). All FTF items are scored as 0, 1 or 2, representing ‘does not apply’ (0), ‘applies sometimes/to some extent’ (1), or ‘definitely applies’ (2). For each domain or subdomain, a mean score of 0–2 was calculated. We converted mean domain/subdomain scores of the parent FTF to z scores (SDSs, standard deviation scores) in relation to the scores for the Swedish age- and gender-specific reference population. Swedish reference normative values for calculating z scores for teacher FTF scales were not available; therefore, we computed z scores for teacher FTF scales relative to the mean scores for control subjects of the same gender. Impairments in individual components/domains of EF and learning skills were defined in terms of SDs that were 2 SD (> 95th percentile) greater than the normative mean in the parent FTF or 2 SD above the mean z scores for the controls in the teacher FTF, corresponding to clinically significant difficulties.

#### Adaptive functioning in school (teacher report)

Assessments of academic performance and adaptive functioning at school were based on the TRF described by Achenbach [34]. Teachers provided responses to the following 5 items on adaptive functioning that measured the child’s performance in academic subjects and 4 adaptive characteristics: ‘How hard is she or he working?’; ‘How appropriately is she or he behaving?’; ‘How much is she or he learning daily?’; and ‘How happy is the pupil?’

#### Demographics

Sociodemographic characteristics, including variables such as the parents’ education level, the family’s disposable income and family structure, were assessed by the Nordic Health and Family Questionnaire [35]. The composite social risk index included maternal education (0 for high school or above and 1 for 9 or fewer years of schooling), family structure (0 for a 2-parent family and 1 for a single-parent family) and family income (0 for high and 1 for low income). The methods used for identification and characterization of NSI, major disability and chronic conditions (NSI and medical or psychiatric illness) in the survivors have been previously described [29,36]. Major NSI was defined as 1 or more of the following: moderate or disabling cerebral palsy (CP); severe visual impairment, including visual acuity < 20/200 without glasses in the best eye or registration at low-vision centers; severe auditory impairment in both ears that could not be corrected with a hearing aid; and hearing loss corrected partially or fully with a hearing aid or an implant. Chronic conditions were defined as NSI and medical or psychiatric illnesses with a duration of 12 months or longer.

### Statistics

Data were collected on standardized forms and encoded for computerized analysis using Windows SPSS 22.0 (SPSS, Chicago, IL). The assessment data for each EI child were examined before they were combined with the data set from 2 previous main studies [3,28] for analysis. Chi-square or Fisher’s exact test for dichotomous outcomes and unpaired *t*-tests for continuous variables were used. Univariate analysis of covariance (ANCOVA) was performed to examine differences between the groups in 1) cognitive components of EF (Similarities, Block design, Picture arrangement, Arithmetic, Coding, and Tower test, and Tower test), 2) the 5 individual domains of behaviors related to EF, 3) adaptive functioning in school, and 4) academic performance. The independent variables in all ANCOVAs were group status (EPT versus control) and gender. Composite social risk score and the mother’s country of birth were entered as covariates. We also performed multivariate analyses of covariance (MANCOVA) to test differences in learning skills between the EPT and control children with FSIQ greater than > 70 (≥ 2 SD above the mean for the normal population according to WISC-III) (31). The independent variables were group status (EPT vs control) and gender, and the outcome variables were each of the 4 subdomains of the learning skills (i.e., reading and writing, math, general learning, and coping in learning) in the parent and teacher FTF. The covariates included in the model were the EFCS, the composite social risk index and FSIQ. MANCOVAs of parent- and teacher-reported learning skills were carried out separately. The effect size (ES) is given by the partial eta-squared statistic, which describes the proportion of total variability attributable to a factor or covariate (the proportion that if multiplied by 100, is the percentage of total variability due to group differences). All significant MANCOVA and ANCOVA effects were interpreted using Cohen’s criteria for ES, where effects are deemed small, medium and large if they account for 1–5.8%, 5.9–13.8% and > 13.8% of variance, respectively [37]. Multivariate logistic regression (MVLR) analyses were carried out to examine differences in the categorical outcomes between the groups after making adjustments for important explanatory variables, namely sex, composite social risk, and the mother’s country of origin. P-values < 0.05 were considered significant.

## Results

### Demographic features

The mean age of assessment for EPT children was 12 (SD: 1.7; range: 10.1–15.6) years, and it was 12.1 (SD: 1.9; range: 10.3–15.9) years for controls. Sociodemographic characteristics collected at the time of the present assessment were similar in the two groups, except that the percentage of mothers of EPT children with an education ≤ 9 years was significantly greater compared with the control group (14% vs 4%, P = 0.04) (Table 1). The EPT children had a mean (SD) birth weight of 718 g (129) and gestational age of 24.4 (0.7) weeks. Fifty-one EPT children (38.6%) had bronchopulmonary dysplasia (BPD); 22 (16.7%) had severe (stage ≥ 3) retinopathy of prematurity; and 9 (6.8%) had grade III-IV intraventricular hemorrhage, periventricular leukomalacia or hydrocephalus with shunt. At the mean age of 12 years, 22 EPT children compared to none in the control group (χ^2^ = 18.9, P < 0.001) had one or more NSI. The corresponding rates of major NSI were 12.9% vs 0 (χ^2^ = 14.3, P < 0.001). The rates of one or more chronic conditions, including NSI or another medical or psychiatric condition, were 53% vs 18.4% (χ^2^ = 29.4, P < 0.001). Twenty EPT children had a diagnosed psychiatric condition according to DSM-IV compared with 2 in the control group (χ^2^ = 12.2, P = 0.001). The 2 most common psychiatric diagnoses were ADHD (n = 11 (8.3%)) and autism spectrum disorder (n = 9 (6.8%)).

**Table 1:** Sociodemographic characteristics, infant birth and neonatal data and chronic conditions at 12 years of age.

	EPT	Control	P value
	N = 132	N = 103	
Maternal age^a^	29.9 (5.3)	30.2 (4.8)	ns
*Adults in home*			ns
Single-parent family, n (%)	19 (14.4)	8 (7.8%)	ns
Two-parent family, n (%)	113 (85.6)	95 (92.2%)	ns
Biological parents, n (%)	80 (60.6)	74 (71.8)	ns
*Non-Nordic country of birth*			
Maternal, n (%)	20 (15.2)	8 (7.8)	ns
Paternal, n (%)	16 (12.3)	11 (10.9)	ns
Both parents, n (%)	12 (9.1)	5 (4.9)	ns
*Maternal education*, *y*			.016
< 9, n (%)	18 (13.6)	4 (3.8)	.04
10–12, n (%)	68 (51.5)	44 (42.7)	ns
>12, n (%)	46 (34.8)	55 (54.5)	ns
*Paternal education*, *y*			ns
< 9, n (%)	15 (12.5)	4 (4.1)	ns
10–12, n (%)	69 (57.5)	55 (56.2)	ns
> 12, n (%)	36 (30)	39 (39.8)	ns
Not known, n	12	4	
*Family income*, *mean (SD)*, *USD*	4399 (1910)	4645 (1608)	ns
Low income, n (%)	40 (27)	27 (26)	ns
Social risk, any^b^, n (%)	50 (37.9)	32 (31)	ns
*Age at assessment*, *mean (SD)*, *y*	11.96 (1.7)	12.09 (1.9)	ns
Range, y	10.1–15.6	10.3–15.9	ns
*Perinatal data*			
Gestational age, mean (SD)	24.4 (0.7)	39.4 (1.29)	< .001
23 weeks, n	16	na	
24 weeks, n	42	na	
25 weeks, n	74	na	
Female, n (%)	72 (54.5)	51 (49.5)	ns
Birth weight, mean (SD), g	718 (129)	3621 (498)	ns
Multiple birth, n (%)	23 (17.4)	na	
SGA^c^, n (%)	21 (15.9)	na	
Born at a tertiary care center, n (%)	132 (100)	132 (100)	
Antenatal steroids, any, n (%)	92 (69.7)	na	
Antenatal steroids, full course	57 (43.2)	na	
Surfactant, n (%)	100	na	
Neonatal survival, n (%)	66	na	
Chronic medical or psychiatric condition at 12 y, ≥ 1, n (%) ^d^^,^ ^e^	70 (53)	19 (18.4)	< .001
Chronic medical condition at 12 y, n (%)^d^^,^^f^	55 (41.7)	17 (16.5)	< .001
Chronic psychiatric condition at 12 y, n (%)^d^	20 (15.2)	2 (1.9)	< .001
Major neurosensory impairment at 12 y, n (%)^d^^,^^g^	17 (12.9)	0	< .001
Full-scale IQ, mean (SD), WISC-III-R	80.3 (18.7)	104.6 (15.7)	< .001

EPT, extremely preterm (23–25 weeks); NSI, neurosensory impairment^;^ ns, not significant; na, not applicable.

^a^ Mother’s age at birth of EI child or control child.

^b^ In calculation of the composite social risk score, 1 point was assigned for each of following: single status, mother’s education of <9 years, and low income.

^c^ SGA indicates ‘small for gestational age’ (birth weight of less than -2 SD) [38].

^d^ Data adapted from ref. no. 36.

^e^ Includes NSI or other medical or psychiatric illness lasting for ≥12 months.

^f^ Includes NSI or other medical illness (excluding psychiatric illness) lasting for ≥ 12 months.

^g^ Includes 1 or more of the following: moderate or disabling cerebral palsy (CP); severe visual impairment, including visual acuity of <20/200 without glasses in the best eye and registration at a low-vision center; severe auditory impairment in both ears not corrected with a hearing aid; and hearing loss corrected partially or fully with a hearing aid or an implant.

### Cognitive assessment of EF

The EPT children scored significantly lower than the controls on all cognitive parameters of EF, including verbal conceptual reasoning (*Similarities*, t _226_ = -6.7, P < 0.001), verbal working memory (*Digit Span*, t _224_ = -7.4, P < 0.001), attention and visual motor integration (*Coding*, t _224_ = -8.2, P < 0.001), working memory and reasoning ability (*Arithmetic*, t _226_ = -9.0, P 0 < .001), spatial conceptualization (*Block design*, t _224_ = -10.2, *P* < 0.001), processing speed, visual reasoning (*Picture arrangement* t _223_ = -6.9, P < 0.001), and planning ability (Tower test, t _224_ = -6.8, P < 0.001) (Table 2). The EPT cohort performed well below the controls; however, the majority of the children were within the normal range. ANCOVAs revealed that significant differences persisted in all cognitive measures of EF between EPT and control subjects when adjustments were made for social risk factors (composite social risk index), the mother’s country of origin and gender. These statistical conclusions did not change when the analyses were carried out in the children with an FSIQ greater than 70 and without major NSIs. However, the magnitude of difference between the groups was narrowed in all cognitive parameters of EF (range 0.66 SD to 1.2 SD) (Table 2). Although social risk was associated with the number of cognitive components of EF, it had a small effect (4–5%) compared to group status, i.e., being EPT (16–31%), in the total population and a medium to large effects (10%–28%) in the children without major NSI and FSIQ > 70 (Table 2). MVLR revealed that EPT children compared to controls had significantly greater clinically significant deficits in all the cognitive areas sensitive to EF when adjustments were made for social risk, gender and the mother’s country of birth (Table 3). When MVLR was carried out on a subgroup of EPT and control children with FSIQ > 70 and without major NSIs, the rates of clinically significant problems (≤ 2 SD) remained significantly higher in 4 areas of EF, including processing speed, non-verbal memory (*Coding*, 8% vs 2%, AOR, 5.5,P = 0.03), working memory, reasoning ability, attention and distractibility (*Arithmetic*, 13% vs 2%, AOR, 7.9, P = 0.008), spatial conceptualization (*Block Design*, 14% vs 0, P < 0.001), and planning ability (*Tower test*, 7% vs 0%, P = 0.007) (Table 3).

**Table 2:** Adjusted mean scores of the EPT and control subjects on cognitive and behavioral parameters of EF.

	Total population				NSI-free and FSIQ > 70			
	EPT	Control	F ratio/P	Effect size^a^	EPT	Control	F ratio/P	Effect size^a^
	N = 125	N = 103			N = 98	N = 99		
*Cognitive assessment*								
Verbal conceptual reasoning (Sim)	9.0 (3.2)	11.7 (2.8)	44.8/< 0.001	0.17	9.9 (2.6)	11.9 (2.7)	28.3/< 0.001	0.13
Verbal working memory (Ds)	7.4 (3.0)	10.2 (2.7)	54.6/< 0.001	0.20	8.1 (2.6)	10.3 (2.6)	34.6/< 0.001	0.15
Processing speed, attention (Co)	7.3 (3.0)	10.5 (2.9)	77.6/< 0.001	0.26	8.0 (2.7)	10.6 (2.8)	57.3/< 0.001	0.23
Attention, memory, distractibility (Ar)	6.5 (2.9)	10.0 (2.9)	82.8/< 0.001	0.27	7.2 (2.6)	10.2 (2.7)	64.2/< 0.001	0.25
Spatial conceptualization (Bd)	6.9 (3.1)	10.9 (2.8)	99.4/< 0.001	0.31	7.7 (2.8)	11.1 (2.6)	75.2/< 0.001	0.28
Visual reasoning (Pa)	7.5 (3.0)	10.3 (3.1)	47.8/< 0.001	0.18	8.2 (2.7)	10.5 (3.0)	32.5/< 0.001	0.15
Planning ability (Tower test)	8.6 (3.3)	11.4 (2.6)	42.4/< 0.001	0.16	9.5 (2.8)	11.5 (2.6)	22.1/< 0.001	0.10
*Behavioral rating of EF*								
Parent report (FTF)								
Executive function composite score^b^	0.51 (0.43)	0.19 (0.23)	49.6/< 0.001	0.18	0.42(0.38)	0.18(0.21)	40.3/< 0.001	0.18
Attention	0.59 (0.50)	0.21 (0.31)	45.6/< 0.001	0.17	0.49 (0.46)	0.17 (0.26)	38.3/< 0.001	0.17
Hyperactivity/impulsivity	0.41 (0.45)	0.16 (0.21)	30.4/< 0.001	0.12	0.33 (0.39)	0.15 (0.21)	20.7/< 0.001	0.10
Hypoactivity	0.42 (0.43)	0.15 (0.27)	30.1/< 0.001	0.12	0.35 (0.39)	0.13 (0.24)	24.8/< 0.001	0.12
Planning/organization	0.72 (0.66)	0.27 (0.40)	39.6/< 0.001	0.16	0.60 (0.56)	0.24 (0.36)	35.2/< 0.001	0.16
Working memory	0.45 (0.47)	0.15 (0.24)	34.1/< 0.001	0.14	0.31 (0.32)	0.12 (0.18)	30.1/< 0.001	0.14
*Teacher report (FTF)*								
Executive function composite score^b^	0.47 (0.44)	0.22 (0.29)	25.4/< 0.001	0.10	0.36 (0.40)	0.20 (0.26)	14.3/< 0.001	0.07
Attention	0.58 (0.57)	0.26 (0.34)	25.7/< 0.001	0.10	0.46 (0.53)	0.24 (0.33)	13.5/< 0.001	0.07
Hyperactivity/impulsivity	0.25 (0.35)	0.17 (0.27)	4.5/0.036	0.02	0.17 (0.31)	0.15 (0.24)	0.65/0.4	0.003
Hypoactivity	0.50 (0.49)	0.21 (0.34)	29.2/< 0.001	0.12	0.42 (0.46)	0.18 (0.31)	21.3/< 0.001	0.10
Planning/organization	0.65 (0.69)	0.24 (0.40)	30.4/< 0.001	0.12	0.46 (0.59)	0.20 (0.35)	15.4/< 0.001	0.08
Working memory	0.47 (0.51)	0.14 (0.27)	37.9/< 0.001	0.15	0.32 (0.38)	0.12 (0.23)	22.4/< 0.001	0.11

The descriptive data are given as the group mean score (SD).

EPT, extremely preterm (23–25 weeks); NSI, neurosensory impairment; Sim, similarities; Ds, digit span; Bd, block design; Pa, picture arrangement; Co, coding; Ar, arithmetic.

The data were obtained using the WISC-III-R [31] and Tower test [30] for the cognitive assessment of EF and using the parent and teacher FTF for the behavioral assessment of EF [32].

The means are adjusted for the children’s gender, social risk (see ‘Methods‘) and maternal country of birth (Nordic vs non-Nordic).

^a^ The effect size is given by the partial eta-squared statistic (see ‘Methods‘).

^b^ For calculation of the executive function composite score, see ‘Methods‘.

**Table 3:** Proportion of children in each group scoring < -2 SD on cognitive parameters of EF.

	Total population				NSI-free and FSIQ > 70			
	EPT	Control	Adjusted OR^a^ (95% CI)	P value	EPT	Control	Adjusted OR^a^ (95% CI)	P value
	N = 128	N = 103			N = 98	N = 99		
Verbal conceptual reasoning (Sim)	17 (13)	0	NA	< 0.001^b^	2 (2)	0	NA	0.24^b^
Verbal working memory (Ds)	26 (20)	2 (2)	12.8 (3–56)	< 0.001	7 (7)	2 (2)	3.6 (.7–19)	0.1
Non-verbal memory, attention (Co)	26 (20)	3 (3)	10.0 (2.9–35.0)	< 0.001	8 (8)	2 (2)	5.5 (1.1–27)	0.039
Memory, attention, distractibility (Ar)	34 (27)	4 (4)	9.1 (3.1–6.2)	< 0.001	13 (13)	2 (2)	7.9 (1.7–37)	0.008
Spatial conceptualization (Bd)	34 (27)	2 (2)	18.0 (4–77)	< 0.001	14 (14)	0	NA	< 0.001^b^
Visual reasoning (Pa)	25 (20)	5 (5)	4.7 (1.8–12.7)	0.003	8 (8)	4 (4)	2.1 (.6–7.3)	0.24
Planning ability (Tower test)	26 (20)	1 (1)	26.0 (3.4–192)	< 0.002	7 (7)	0	NA	0.007^b^

EPT, extremely preterm (23–25 weeks); NSI, neurosensory impairment; FSIQ, full-scale intelligence quotient; Sim, similarities; Ds, digit span; Bd, block design; Pa, picture arrangement; Co, coding; Ar, arithmetic; OR, odds ratio; CI, confidence interval; NA, not appropriate for calculation due to wide confidence intervals for exponents.

The values are n (%) unless otherwise stated. The data were adapted from core subtests of the WISC-III-R (Swedish edition, Wechsler 1999) [31] and the Tower test of the Delis-Kaplan Executive Function Scale [30].

^a^ The ORs were derived from logistic regression analyses adjusted for the children’s gender and social risk (any vs none, see ‘Methods‘) and mother’s country of birth (Nordic vs non-Nordic).

^b^ P values were determined by Fisher’s exact test for the EPT children vs control subjects.

### Behavioral parameters related to EF by group and by evaluation source

#### Psychometric analysis

A high level of reliability was noted in the analyses of behaviors related to EF, working memory, and learning skill domains, as well as in the subdomain scores in teacher and parent FTF. Cronbach’s alpha for the domain scores (items) in the teacher and parent FTF, respectively, were 0.96 and 0.95 for the executive function domain, 0.96 and 0.97 for the learning skills domains, and 0.93 and 0.94 for the memory domain. The correlations between the subdomain scores from teacher and parent FTF ranged from r = 0.52–0.81 and r = 42–0.78, respectively. Most of the subdomain scores of the teacher and parent FTF moderately correlated with one another, with *r* values between 0.34 and 0.82 (all rs, P < 0.001). The correlations between the scores for the cognitive aspects of EF (Similarities, Digit span, Block design, Picture arrangement, Coding, Arithmetic and Tower test) and the executive function composite score (EFCS) from the parent and teacher FTF ranged from r = 0.40–0.48 and from r = 0.45–0.55, respectively (all rs P <0.001), indicating moderate correlations between the cognitive aspects of EF and behaviorally defined EF.

#### Behavioral parameters related to EF

The mean raw EFCS scores were significantly higher in EPT children compared with the controls, indicating an increased risk for executive dysfunctions (t = 6.7, P < 0.001 for parent FTF and t = 4.74, P < 0.001 for the teacher FTF). Compared to the controls and according to both the parent and teacher reports, EPT children had significantly higher mean scores for all 5 individual behavior subdomains related to EF indicating an increased risk for problems with these behaviors (Table 4). The ANCOVA revealed that the significant differences persisted between the EPT and control groups after adjusting for social risk factors (composite social risk index), gender and maternal country of birth (Nordic vs non-Nordic). Although the magnitude of the difference between the groups decreased, the statistical conclusion was not altered when ANCOVA was carried out in a subgroup of EPT and control children with FSIQ > 70 and without major NSIs (Table 2), indicating the persistence of a higher risk for behavioral problems related to EF in EPT children. Social risk was associated with a number of behaviors related to FTF; however, they represented small effects (4% to 5%). Compared with those of controls, parents and teachers of EPT children were more likely to rate their child as scoring in the clinical range (> 2 SD) for a number of individual subdomains of behaviors related to EF (Table 4). The teacher-reported rates of clinically significant problems among total and NSI-free EPT children with FSIQ > 70, respectively, were for attention, 26% (AOR 5.6, P < 0.001) and 18% (AOR 4.2, P = 0.007); for hypoactivity, 20% (AOR 5.0, P = 0.002) and 16% (AOR 6.3,P = 0.005); for planning and organizing, 26% (OR 8.6, P < 0.001) and 16% (OR 6.7, P = 0.004); and for working memory, 27% (AOR 9.6, P < 0.001) and 16% (AOR 9.9,P = 0.003) (Table 4). Conversely, teachers did not rate EPT children as significantly different in hyperactivity/impulsivity behaviors related to EF.

**Table 4:** Proportion of children in each group scoring in the clinical range (> 2 SD above the mean) on behavioral parameters of EF.

	Total population			NSI-free and FSIQ > 70		
FTF scale	EPT	Control	Adjusted OR^a^ (95% CI)/P	EPT	Control	Adjusted OR^a^ (95% CI)/P
	N = 128	N = 103		N = 98	N = 99	
*Parent report (FTF)*						
Executive function composite score	18 (14.3)	1 (1)	16.1 (2.1–122.1)/0.007	8 (8.2)	0	NA/0.003^b^
Attention	16 (12.6)	1 (1)	13.5 (1.8–104.0)/0.013	9 (9.2)	0	NA/0.002^b^
Hyperactivity/impulsivity	14 (11.1)	0	NA/<0.001^b^	7 (7.2)	0	NA/0.007^b^
Hypoactivity	16 (12.6)	3 (2.3)	4.4 (1.2–15.7)/0.023	9 (9.2)	1	10.7 (1.3–89.9)/0.029
Planning/organization	34 (26.9)	7 (6.8)	4.6 (1.9–10.9)/0.001	16 (16)	5 (5)	3.3 (1.2–9.6)/0.027
Working memory	25 (19.8)	4 (3.8)	5.6 (1.9–16.8)/0.002	9 (9.2)	1	10.2 (1.3–83.2)/0.03
*Teacher report (FTF)*						
Executive function composite score	29 (23.7)	5 (5)	5.7 (2.1–15.4)/0.001	15 (15)	3 (3)	5.8 (1.6–21.1)/0.007
Attention	33 (25.8)	6 (5.9)	5.6 (2.2–14.0)/< 0.001	18 (18)	5 (5)	4.2 (1.5–11.9)/0.007
Hyperactivity/impulsivity	17 (13.3)	6 (6.1)	2.6 (.95–67.0)/0.06	8 (8.2)	5 (5)	1.8 (.52–6.0)/0.35
Hypoactivity	26 (20.3)	5 (5.1)	5.0 (1.8–13.8)/0.002	16 (16.2)	3 (3)	6.3 (1.8–22.4)/0.005
Planning/organizing	33 (25.8)	4 (4.1)	8.6 (2.9–25.4)/< 0.001	16 (16.2)	3 (3)	6.7 (1.8–24.2)/0.004
Working memory	34 (26.5)	4 (3.9)	9.6 (3.3–28.6)/< 0.001	16 (16.2)	2 (2)	9.9 (2.1–45.0)/0.003

EPT, extremely preterm (23–25 weeks); NSI, neurosensory impairment; NA, not appropriate for calculation due to wide confidence intervals for exponents.

The values are n (%) of children unless otherwise stated.

Data were obtained from the parent and teacher FTF questionnaires [32].

^a^ The ORs were derived from logistic regression analyses adjusted for the children’s gender, social risk, and maternal country of birth (Nordic vs non-Nordic).

^b^ P values were determined by Fisher’s exact test for the EPT children vs control subjects.

### Adaptive functioning in school

On the TRF scale that assessed academic performance in children with FSIQ > 70 and without major NSI, EPT children had significantly poorer scores than control participants (mean difference, -0.67, t _190_ = -7.2, P < 0.001) (Table 5). ANCOVA revealed that the group status (prematurity) had a large effect, accounting for 21% of the variance, indicating that prematurity was strongly associated with poor academic performance. None of the other covariates were associated with the academic performance of either EPT or control children. Furthermore, the EPT children had significantly poorer scores than the control children with respect to TRF ratings of total adaptive function, which was computed by summing ratings for 4 adaptive characteristics (t _190_ = -5.5, mean diff, -2.94, P < 0.001) (Table 5). In the domain of ‘how satisfied and happy a child is’ the two groups were not rated differently. There were no significant interactions between gender and group regarding these variables.

**Table 5:** Mean scores for adaptive functioning, academic performance and learning skills, as assessed by teacher and parent reports, in each group without major NSI and with FSIQ > 70.

	Major NSI-free and FSIQ > 70^a^			
Items	EPT	Control	F-ratio/P	Effect size
	N = 98	N = 99		
*Adaptive functioning scale (TRF)*				
Academic performance	2.9 (0.60)	3.6 (0.7)	50.1/<0.001	0.21
Works hard	4.0 (1.4)	4.9 (1.1)	29.7/<0.001	0.14
Behaves appropriately	3.9 (1.1)	4.7 (1.2)	20.9/<0.001	0.10
Learns in class	3.8 (1.1)	4.9 (1.2)	44.2/<0.001	0.19
Happy and satisfied	4.5 (1.0)	4.8 (1.0)	3.4/0.06	0.02
Total adaptive score^b^	16.3 (3.46)	19.2 (3.87)	33.9/<0.001	0.15
*Learning skills*, *teacher report (5–15)*				
Reading/writing	0.41 (0.52)	0.15 (0.29)	19.6/<0.001	0.10
Math	0.70 (0.66)	0.13 (0.34)	58.4/<0.001	0.24
General learning	0.57 (0.44)	0.30 (0.30)	27.7/<0.001	0.13
Coping in learning	0.50 (0.51)	0.15 (0.26)	37.1/<0.001	0.16
*Learning skills*, *parent report (5–15)*				
Reading/writing	0.33 (0.44)	0.14 (0.26)	20.3/<0.001	0.10
Math	0.68 (0.64)	0.13 (0.28)	56.5/<0.001	0.23
General learning	0.86 (1.1)	0.38 (0.76)	12.3 /0.001	0.06
Coping in learning	0.48 (0.45)	0.16 (0.25)	42.6 /<0.001	0.18

EPT, extremely preterm (23–25 weeks); NSI, neurosensory impairment; FSIQ, full-scale intelligence quotient.

The data were adapted from the adaptive functioning scale of the TRF [34] and from the FTF parent and teacher questionnaires [32].

The means were adjusted for the children’s gender, social risk and maternal country of birth (Nordic vs non-Nordic).

The effect size is given by the partial eta-squared statistic (see ‘Methods‘ for description).

^a^ FSIQs were determined using the WISC-III [31].

^b^ The total adaptive score was derived from the sum of the following 4 adaptive characteristics from the TRF adaptive functioning scale [34]: ‘How hard is she or he working?’; ‘How appropriately is she or he behaving?’; ‘How much is she or he learning daily?’; and ‘How happy is the pupil?’

### Learning skills

We compared EPT children and controls (EPT 98; control, 99) with FSIQ > 70 regarding the four subdomains of learning skills. According to both parent and teacher FTF, the EPT cohort had significantly higher scores in all subdomains of learning skills, indicating an increased risk of learning problems in reading/writing, math, general learning and coping in learning (Table 6). ANCOVA revealed that significant differences persisted between the two groups when adjustments were made for composite social risk, gender and mothers’ country of birth (Nordic vs non-Nordic). Group status (prematurity) had a medium to large effect (range, 10% to 24% in teacher FTF and 6% to 23% in parent FTF) (Table 5). As shown in Table 6, according to teacher FTF, the EPT cohort with FSIQ >70 and without major NSIs was at a significantly increased risk of having clinically significant problems in math (37% vs 6%, AOR, 8.8, P < 0.001), reading/writing (17% vs 6%, AOR 3.6, P = 0.012), general learning (15% vs 1%, AOR 18.2, P = 0.006) and coping in learning (25% vs 5%, AOR 6.3, P < 0.001). Parents rated learning problems similarly, but the proportion of EPT children with clinically significant problems in all 4 learning skills domains was lower than that in the teacher reports. The most prevalent learning problem was in math according to both teacher and parent reports. In a separate analysis carried out in children with FSIQ > 85 and without major NSI, the EPT children still had clinically significant problems in math (16% vs 2%; AOR, 7.9, P < 0.001).

**Table 6:** Proportion of children in each group with FSIQ > 70 and without major NSI scoring in the clinical range for learning skill problems (>2 SD) on the parent and teacher FTF.

	NSI-free and FSIQ > 70			
	EPT	Control	Adjusted OR (95% CI)^a^	P value
	N = 98	N = 99		
*Parent report (5–15)*				
Reading/writing	12 (12)	1 (1)	12.5 (1.6–99.1)	0.017
Math	17 (17)	1 (1)	21.4 (2.8–165.2)	0.003
General learning^b^	12 (12)	0	NA	< 0.001
Coping in learning	13 (13)	1 (1)	15.0 (2.0–117)	0.010
*Teacher report (5–15)*				
Reading/writing	17 (17)	6 (6)	3.6 (1.3–9.7)	0.012
Math	35 (37)	6 (6)	8.8 (3.5–22.2)	< 0.001
General learning	14 (15)	1 (1)	18.2 (2.3–142.6)	0.006
Coping in learning	24 (25)	5 (5)	6.3 (2.3–17.6)	< 0.001

EPT, extremely preterm (23–25 weeks); NSI, neurosensory impairment; FSIQ, full-scale intelligence quotient; OR, odds ratio; CI, confidence interval; NA, not appropriate for calculation due to wide confidence intervals for exponents.

The values are n (%) unless otherwise stated. The data were adapted from the learning skills domains of the parent and teacher FTF [32].

^a^ The ORs were derived from logistic regression analyses adjusted for the children’s gender, social risk and mother’s country of birth.

^b^ P value was determined by Fisher’s exact test for the EPT children vs control subjects.

### MANCOVA effects by teacher report (FTF)

The 2 (group status) x 2 (gender) MANCOVA of 4 teacher FTF learning scales revealed significant multivariate effects of EFCS ([Wilks **λ** = 0.41], F_4_,176 = 64.0, P < 0.001, ES = 59%); group status (prematurity vs control) ([Wilks λ] = 0.90, F_4_,176 = 5.1, P = 0.001, ES,10%); and FSIQ ([Wilks λ = 0.93], F_4_,176 = 3.4, P = 0.01, ES = 7%).

### MANCOVA effects by parent report (FTF)

As with the teacher report, MANCOVA of the parent FTF learning scales revealed similar effects of EFCS ([Wilks λ] = 0.47, F_4_,179 = 50.1, P < 0.001, ES,53%); group status (prematurity vs control) ([Wilks λ] = 0.95, F_4_,179 = 4.5, P = 0.003, ES 7%); and FSIQ ([Wilks λ] = 0.89, F_4_,179 = 5.3, P < 0.001, ES,11%). No interactions emerged in the MANCOVA of parent and teacher reports. These analyses indicated that in the study of children without significant NSI and a normal or low to normal IQ, executive dysfunctions had a strong association with learning problems in school, and these effects were mediated independently beyond the FSIQ.

## Discussion

In this study, relying on measures that assessed cognitive faculties sensitive to EF and relying on teachers and parents’ behavioral ratings related to EF, the EPT children in their early adolescence had a significantly increased risk for problems in measures of executive function, even those without significant NSI and with a normal or low-normal overall intelligence.

Our study focused on the cognitive assessment of EF, including verbal conceptual reasoning, spatial conceptualization, working memory, attention, planning and organizational ability. The EPT children exhibited impairments in all of these EF measures. The magnitude of mean differences between the EPT children and controls averaged to 1.1 SD for cognitive parameters (0.9–1.16 SD) in the total population. However, the group means of EPT children were generally within the normal or low normal range, and the proportion of EPT children who exhibited clinically significant impairments in the cognitive parameters of EF was relatively small (13–27%). When the children with major NSI and FSIQ < 70 were excluded, a significantly higher number of EPT children with clinically significant impairments remained, selectively in the cognitive areas sensitive to EF, such as spatial conceptualization, working memory, attention, processing speed, and planning ability. Similar to the findings from other studies [12,16,20], our data indicate that deficits over a wide range of EF that have been reported in cohorts of very preterm newborns at elementary and middle school age [12–19] persist into adolescence. It is important to identify in early childhood the cognitive deficits sensitive to EF, as research has shown that mild to moderate deficits in EF have a significant impact on learning skills and scholastic attainment [10,19,20,23,24]. Impairments in EF translate into behavioral difficulties in school and at home as reported by parents and teachers [12,16,18,39]. In our EPT cohort, both parents’ and teachers’ ratings displayed a significantly increased risk for behavioral problems related to EF. A significantly higher proportion of EPT children compared with controls displayed clinically significant difficulties in attention, hypoactivity, planning/organizing and working memory. Conversely, parents but not teachers reported increased rates of clinically significant hyperactivity/impulsive behavior in the EPT group compared to controls, a finding similar to those in other studies [18,39–41]. This result is not surprising, as each rater can provide important and different information on a child’s behavior because perceptions and interpretations of behavior vary and may be valid reflections of the child’s behavior in different contexts [42–44]. As in other research [12,18,20], our findings suggest that EPT children without significant NSI and with FSIQ in the normal range have an increased risk for mild to moderate problems in behaviors related to EF. This outcome affects learning skills and academic attainment (see later discussion). It is concerning that the children who exhibit mild or moderate problems are less likely to arouse parental or teacher concerns, to be referred for assessment and to receive specialist assistance at the appropriate time.

The overall findings in our study were robust, and they were not affected when adjusting for important socioeconomic variables, gender, and maternal ethnicity. Likewise, statistical conclusions were largely unaltered when children with major NSI and FSIQ < 70 were excluded from the analyses. The correlational (univariate) analyses (data not shown) revealed that gestational age and birth weight were weakly correlated with EF parameters, both behavioral and cognitive, indicating that other factors must affect the outcomes. It is noteworthy that the cognitive deficits exhibited by our EPT cohort were not specific to EF. When compared with the control group, EPT children had deficits in nonexecutive skills, such as general knowledge (information subtest, mean difference, -2.5; 95% CI: -3.3 to -1.8), vocabulary (vocabulary subtest: -2.1; 95% CI: -2.8 to -1.3), and visual reasoning (picture completion subtest: mean difference: -2.4; 95% CI -3.3 to -1.6). Thus, commensurate with other studies [6,7,13,16,40,45,46], EPT children were at an increased risk for impairment across a range of cognitive faculties, not only those that were sensitive to EF. The cognitive and educational problems in EPT children are thought to be associated with several risk factors, such as brain injury (periventricular leukomalacia) [47], disruptions in the cortical development in the absence of concomitant biomedical risk [48], nutritional problems, metabolic complications in the neonatal period and environmental stresses [49]. It is not clearly understood whether executive dysfunctions in EI children are a primary deficit or secondary to deficits in the other cognitive domains, and this knowledge gap may be better resolved with neuroimaging studies.

We did not control for IQ in analysis of the group differences in the cognitive aspects of EF or in the behaviors related to EF. However, we did control for IQ in multivariate analyses of learning skills in the children with FSIQ >70 and in those without major NSI. An important limitation of analyses of IQ-adjusted scores is that the demands of specific measures such as executive functions may be similar in some of the subsets contributing to IQ. In addition, it has been argued that controlling for IQ when assessing group differences in scores for EF measures and motor ability removes the very same variance that one is interested in studying, resulting in decreased power for detecting group differences [50]. Thus, using IQ as a matching variable or covariate may produce overcorrected, anomalous, and counterintuitive findings regarding neurocognitive functions [51].

We examined the differences in learning skills between EPT and control children with FSIQ > 70 and without major NSIs. In the multivariate models, we controlled for important variables, such as EFCS, FSIQ, gender, social risk factors, and mothers’ country of birth. Although group status and FSIQ were independently related to an increased risk for learning problems, EFCS, which is a composite measure of behaviors related to EF, had a much stronger effect explaining 53% and 59% of the variance according to teacher and parent reports, respectively. Based on these findings, we can reasonably conclude that 1) mild to moderate problems in the behaviors related to EF have a negative impact on learning skills and 2) these large effects are mediated beyond IQ. The most prevalent difficulty was observed in math, according to teacher reports (37%) and parent reports (17%), in EPT children with FSIQ > 70. Even in a subgroup of children with normal IQs of > 85, these rates were 16% vs 2% (AOR 7.9, 95% CI 1, 4–42, 9, P < 0.001) according to teacher ratings. This finding is consistent across studies that have shown that problems in math are independent of FSIQ [23,43,46,52–54]. Few studies have identified EF as a set of skills for achievement in math in extremely or very preterm children [17,20,55,56]. As discussed earlier, in the EPT cohort, even children with intelligence in the normal or lower normal range and without major NSI had increased risk for deficits in spatial conceptualization, planning/organizing and verbal/spatial memory. These are the cognitive areas that have been linked to domain general predictors of math difficulty in preterm children [17,20,24]. In an attempt to specify the area of deficit in math skills, very few studies have suggested an association with one of the domain-specific predictors of math skills, namely basic numerical processing [57–60]. However, the results are conflicting [61]. More research is needed to examine the role of specific predictors, particularly in combination with general predictors in relation to mathematical skills.

There are some limitations of this study that affect the generalizability of our findings. First, our study is not population-based, and it reflects the practices of 2 perinatal referral centers that have had consistent policies of active perinatal care since the beginning of the 1990s [62]. However, 82% of all EPT children born alive in the 2 northern regions during the study period were born at these 2 centers, indicating a very high degree of centralization [3,28]. Second, the use of other specific tests of EF would have added substantial information. However, we had to make a selection of methods, and we assumed that the addition of behavioral correlates to EF would further strengthen the study. Our data supports this assumption by demonstrating significant correlations between the cognitive aspects of EF and behaviorally defined EF. Third, the parents and teachers were not blinded to the group status when answering the FTF and TRF. However, the psychometric properties of these questionnaires have been shown to be good [32].

The important strengths of this study are the very high rate of follow-up (98% of all survivors), the use of a relevant control group, and the acquisition of information on the study children’s daily functioning from both parents and teachers. A further strength of the study is that we used a valid instrument focusing on behavioral problems related to EF and learning skills. The FTF questionnaire has been in use for almost a decade in Scandinavian countries for both clinical and research applications. The parts of the FTF that assess fine motor skills, EF, perception and language correlate significantly with the corresponding NEPSY domain scores (A developmental neuropsychological assessment) [63]. The parent FTF has also shown fairly good validity against the WISC-III developmental test [64].

## Conclusion

Adolescent children born at the limit of viability have an increased risk of impairments in their executive functions and behaviors related to them. Deficits in EF remain, even in children with low normal or normal intelligence and without major NSIs, which partly explains their lower scholastic attainment and deficient learning skills. Neuroimaging techniques will make it possible to identify the underlying executive processes in EPT children. This approach may allow for exploring the interventions needed for the development of compensatory functional circuitry. Knowledge of the course of neuropsychological impairments, including EF in early childhood and beyond, is crucial in identifying the need for intervention strategies. Another important implication of studies similar to ours is that there is an urgent need to convey this information to stakeholders, i.e., parents, caretakers and teachers, so that children requiring referrals, diagnostic procedures and interventions are picked up early in the course. Thus, a long-drawn negative developmental cascade on learning may be moderated or prevented.
